# Comprehensive In Silico Analysis Identifies MSTO1 and LIG1 as Candidate Biomarkers With Diagnostic and Prognostic Relevance in Hepatocellular Carcinoma

**DOI:** 10.1002/cnr2.70685

**Published:** 2026-09-13

**Authors:** Jana Hamad, Hussein Fayyad‐Kazan, Fadi Abdel‐Sater

**Affiliations:** ^1^ Faculty of Sciences I, Laboratory of Cancer Biology & Molecular Immunology Lebanese University Beirut Lebanon

**Keywords:** bioinformatics, biomarkers, HCC, LIG1, MSTO1

## Abstract

**Background:**

Hepatocellular carcinoma (HCC) is the most common primary liver malignancy and remains a major cause of cancer‐related mortality worldwide. Its poor clinical outcomes are largely attributed to late‐stage diagnosis and the limited accuracy of currently available diagnostic and prognostic biomarkers. Therefore, identifying novel molecular markers with improved sensitivity, specificity, and therapeutic relevance is essential for enhancing early detection and guiding personalized treatment strategies.

**Aims:**

To identify and prioritize novel candidate HCC biomarkers with diagnostic and prognostic value and potential therapeutic vulnerability using integrated multi‐omics, survival, functional dependency, and tumor microenvironment analyses.

**Methods and Results:**

We examined the mRNA and protein expression levels of 8 DEGs in HCC tissues in the TCGA and CPTAC datasets using UALCAN, which showed that MSTO1 and LIG1 were overexpressed consistently in HCC relative to normal liver tissues. Moreover, elevated expression levels of these genes were significantly associated with higher tumor grade and advanced stage. Kaplan–Meier plotter survival data confirmed that increased expression of MSTO1 and LIG1 was associated with poorer overall survival. The DepMap CRISPR knockout data confirmed a functional dependency of both genes in HCC cell lines. CBioPortal analyses provided characterization of genomic alterations and enabled enrichment analysis of co‐expressed genes, and the TCGA‐UALCAN pan‐cancer analyses supported the assessment of tissue specificity across tumor types. TIMER3 analyses linked candidate gene expression with immune cell infiltration patterns. Diagnostic performance by ROC analysis showed excellent discrimination for MSTO1 (AUC = 0.987) and good discrimination for LIG1 (AUC = 0.897). Multivariate Cox regression with Benjamini‐Hochberg FDR correction across the eight genes supported MSTO1 as a candidate independent prognostic factor after adjustment for tumor stage, grade, etiology, age, and sex (HR = 1.29, *p* = 0.035), whilst LIG1 showed no independent prognostic value. Promoter methylation of MSTO1 and ADH4, assessed via UALCAN, showed that both genes were significantly differentially methylated in the promoter region of primary HCC tissues compared with normal liver tissues. Our study also confirmed the biological and clinical relevance of established HCC biomarkers: TERT, IRAK1, and ADH4.

**Conclusion:**

MSTO1 and LIG1 emerged as candidate diagnostic biomarkers in HCC. Additionally, MSTO1 showed a candidate prognostic association with overall survival that remained significant after adjusting for tumor stage, grade, and etiology, as well as patients' age, but not after further adjustment for AFP status. Functional data also highlighted MSTO1 as a candidate therapeutic dependency. On the other hand, LIG1 showed no independent prognostic association in either multivariate model. Their differential expression and functional essentiality in HCC cell lines highlighted their value for further experimental and independent‐cohort validation before potential integration into biomarker development pipelines aimed at improving early detection and targeted therapy in HCC.

## 1 Introduction

Hepatocellular carcinoma (HCC), the most common primary liver cancer arising from hepatocytes, represents a major global health challenge. It is currently the sixth most prevalent cancer and the third leading cause of cancer‐related mortality worldwide, accounting for approximately 757 948 deaths annually [1]. Its incidence increases markedly with advancing age and occurs three times more frequently in men than in women [1, 2]. HCC is a highly heterogeneous tumor, reflecting its diverse etiologies, and continues to demand advances in pathogenesis, diagnostics, prevention, therapeutics, and personalized medicine [3]. It is strongly associated with chronic liver disease and cirrhosis, with the predominant etiological factors being chronic hepatitis B virus (HBV) and hepatitis C virus (HCV) infections, excessive alcohol intake, and aflatoxin B1 exposure. Additional contributors include non‐alcoholic fatty liver disease (NAFLD), non‐alcoholic steatohepatitis (NASH), diabetes, hereditary hemochromatosis, α1‐antitrypsin deficiency, porphyria cutanea tarda, hereditary tyrosinemia, and long‐term oral contraceptive use [4, 5, 6, 7, 8, 9].

The management of HCC has evolved, but the clinical landscape remains challenging. Alpha‐fetoprotein (AFP) remains the most widely used biomarker for HCC diagnosis and prognosis. Although its sensitivity and specificity are limited, elevated AFP levels may precede clinical diagnosis by several months [10]. Its glycosylated isoform, AFP‐L3, shows greater specificity because it is produced exclusively by HCC cells. Other protein biomarkers investigated in HCC include des‐gamma‐carboxy prothrombin (DCP) [10], alpha‐L‐fucosidase (AFU) [11], and glypican‐3 (GPC3) [12].

Treatment options for HCC are stage‐dependent, ranging from surgical resection, liver transplantation, and local ablation in early stages to trans‐arterial chemoembolization (TACE) and systemic therapies in advanced stages [13]. For advanced HCC, sorafenib, a multikinase inhibitor targeting Raf kinase and angiogenic pathways, was the first systemic agent to demonstrate a survival benefit and became the standard first‐line therapy worldwide [14]. Recently, immunotherapy has become particularly promising, with immune checkpoint inhibitors (ICIs) such as nivolumab (anti–PD‐1) and pembrolizumab (anti–PD‐1) demonstrating clinical benefit [15], and the combination of atezolizumab (anti–PD‐L1) with bevacizumab (anti–VEGF) showing improved survival compared to sorafenib [13, 16]. Further combinations, including nivolumab with ipilimumab (anti–CTLA‐4) [17, 18] and durvalumab (anti–PD‐L1) with tremelimumab (anti–CTLA‐4) [14], have expanded therapeutic options, underscoring the growing role of immunotherapy in HCC management. Despite these advances, durable responses were achieved in only a minority of patients, underscoring the complexity of tumor‐immune interactions [14]. To address these therapeutic and diagnostic challenges, there is a need for novel tumor‐associated antigens that can be used as specific biomarkers for patient stratification.

The rapid development of in silico methodologies has provided a powerful solution to this challenge by enabling efficient, large‐scale analysis of genomic and transcriptomic datasets, including those from The Cancer Genome Atlas (TCGA). These computational approaches bridge the gap between raw molecular data and clinical application, enabling investigators to go beyond traditional biomarkers and pinpoint the molecular drivers of HCC progression. This method not only improves reproducibility and decreases the time and cost of experimental validation but also enables the identification of targets important for personalized healthcare. By using open‐access databases, they also promote cross‐institutional collaboration and comparative analyses between normal and tumor tissues across various cancer types, advancing diagnosis and prognosis, informing personalized treatment strategies, and facilitating biomarker discovery to improve patient outcomes.

To create a balanced discovery and validation platform, we picked eight candidate genes from the first set of differentially expressed genes (DEGs) that included both well‐known oncogenic benchmarks and novel, poorly characterized targets. To validate the robustness of our analytical pipeline, we included well‐characterized HCC markers such as TERT, IRAK1, and ADH4 as internal positive controls and found these to be significantly deregulated in our data, confirming the biological validity of our strategy. We also focused on highly significant transcripts that are significantly dysregulated in HCC but functionally poorly characterized in the literature of liver malignancy, such as MSTO1 and LIG1. The objective of this study was to assess this curated set of validated clinical benchmarks and new candidates, validate a robust multi‐gene framework for HCC assessment, and identify previously unrecognized molecular drivers with potential diagnostic and prognostic utility.

## 2 Methods

### 2.1 Criteria for Candidate Gene Selection

Three types of selection criteria were used to screen the initial pool of differentially expressed genes (DEGs) to the final eight candidate targets, using the UALCAN portal:

– *Statistical Robustness*: Inclusion of targets required highly significant transcriptomic and proteomic dysregulation (*p* < 0.05) generated by UALCAN with clear directionality, establishing a panel of four upregulated and four downregulated genes.
– *Methodological Benchmarking*: Incorporation of well‐established HCC oncogenic drivers (e.g., TERT, ADH4, and IRAK1) as internal positive controls to demonstrate the technical integrity and biological accuracy of the analytical pipeline.
– *Functional Novelty and Biological Rationale*: Prioritization of significantly dysregulated transcripts that are conspicuously missing or poorly characterized in the context of liver malignancy literature (MSTO1, LIG1, GHR, ASPG, and HBB). All of these novel candidates have defined biological significance that may be related to tumor progression. MSTO1 regulates mitochondrial dynamics and cellular morphology and may be associated with cancer cell progression; LIG1 is required for DNA replication and repair and responds to replication stress in rapidly proliferating malignancies; GHR regulates growth factor signaling axes involved in tumor cell proliferation; ASPG plays a role in cellular amino acid metabolism and stress adaptation; and HBB mirrors important changes in tumor oxygenation and microenvironmental stress responses. We demonstrate untapped clinical utility of these under‐reported molecular features for diagnostics and prognosis in HCC.

To provide direct quantitative support for the statistical robustness criterion above, log2 fold‐change and significance were independently computed for each of the eight final candidate genes using the same TCGA‐LIHC RNA‐seq expression data (HCC tumor, *n* = 371; adjacent normal liver, *n* = 50) used for the ROC/AUC analyses described below. For each gene, log2 fold‐change was calculated as the difference between mean log2‐transformed tumor and normal expression, and statistical significance was assessed using an unpaired Welch's *t*‐test (unequal variances; SciPy v1.18.0); resulting *p*‐values were adjusted for multiple testing across the eight genes using the Benjamini‐Hochberg false discovery rate (FDR) procedure (NumPy v2.5.1, Python v3.12).

### 2.2 UALCAN

The Cancer Genome Atlas Liver Hepatocellular Carcinoma (TCGA‐LIHC) data accessed in 2026 were accessed through the Xena browser (https://xenabrowser.net/) and the University of ALabama at Birmingham CANcer data analysis (UALCAN) portal. The UALCAN online portal (http://ualcan.path.uab.edu) was used to examine the distribution of the mRNA expression levels of our candidate upregulated (LIG1, TERT, IRAK1, and MSTO1) and downregulated (ADH4, GHR, ASPG, and HBB) genes in normal liver tissues versus HCC tissues, cancer grades, and stages. It was also used to analyze their protein expression (CPTAC data, mass spectrometry technique, accessed 2026) levels and pan‐cancer expression profile.

### 2.3 Human Protein Atlas (HPA)

The Human Protein Atlas (https://www.proteinatlas.org/, HPA version 23.0) was used to assess the relative protein expression of our candidate genes in normal liver tissues and HCC tissues based on immunohistochemistry data derived from tissue microarrays (TMA).

### 2.4 Kaplan–Meier Plot

Survival analysis was performed using the UALCAN online platform. UALCAN generates Kaplan–Meier survival plots to evaluate the prognostic impact of gene expression in HCC. These plots represent overall survival (OS), and patients were automatically stratified into high‐ and low‐expression groups for each gene analyzed.

### 2.5 Cox Multivariate Analysis

To assess whether MSTO1 and LIG1 were independently associated with overall survival, multivariate Cox proportional hazards regression was performed (lifelines v0.30.3, Python, Efron approximation) on TCGA‐LIHC clinical and expression data, adjusting for patient age, sex, and the American Joint Committee on Cancer (AJCC) pathological stage, histologic grade and etiology (Model 1, *n* = 325), in addition to serum AFP status (Model 2, *n* = 254; etiology dropped due to combined data missingness). Because eight candidate genes were evaluated for a survival association, the resulting log‐rank *p*‐values were additionally corrected for multiple testing using the Benjamini‐Hochberg false discovery rate (FDR) procedure. Exploratory subgroup analyses of MSTO1 expression and survival were performed within etiology categories using the log‐rank test.

### 2.6 Cancer Dependency Map (DepMap)

*DepMap* (https://depmap.org/portal/, 26Q1)‐ CRISPR knockout data were used, using UALCAN, to assess the functional dependency potential of TERT, LIG1, IRAK1, MSTO1, ADH4, GHR, ASPG, and HBB in 23 liver cancer cell lines, providing insight into which genes are critical for HCC cell survival and may represent potential therapeutic vulnerability.

### 2.7 Cbioportal

Genomic alterations in TERT, MSTO1, LIG1, IRAK1, and ADH4 were analyzed using cBioPortal for Cancer Genomics (https://www.cbioportal.org/, v7.0.0), an open‐access platform that integrates multidimensional cancer genomics data from TCGA and other large‐scale studies. The TCGA‐LIHC dataset was selected to assess the frequency and type of alterations, including mutations, copy number variations, and amplifications. Cbioportal was also used for analyzing the first 100 significant genes co‐expressed with MSTO1 and LIG1 for enrichment pathway analysis of these genes.

### 2.8 Epigenetic Regulation Analysis

We analyzed promoter methylation profiles of MSTO1 and ADH4 in the TCGA‐LIHC dataset through the UALCAN portal to investigate potential epigenetic regulation. Promoter methylation levels were quantified using DNA methylation *β*‐values (0 = unmethylated, 1 = fully methylated). This analysis allowed us to correlate the promoter methylation status with the transcriptional changes observed for our candidate genes in HCC.

### 2.9 Receiver Operating Characteristic (ROC) Analysis

ROC curve analysis was performed to evaluate the diagnostic potential of the identified candidate genes using GraphPad Prism 10, using mRNA expression data from TCGA in HCC and normal liver tissue samples. The diagnostic accuracy of each candidate gene (MSTO1, TERT, LIG1, IRAK1, ADH4, GHR, ASPG, HBB, and AFP) was evaluated using the Area Under the Curve (AUC), with the diagonal line (AUC = 0.5) as a baseline for random classification. Performance indicators, including AUC values, standard errors, *p* values, and 95% confidence intervals (CIs), were calculated for each gene to assess sensitivity and specificity and to determine its statistical significance.

### 2.10 Association Between Gene Expression and Immune Cell Infiltration

Correlation of MSTO1 and LIG1 expression with the amount of infiltration of various immune cells was explored to examine the potential effect of these genes on the HCC tumor microenvironment. The association between these parameters was assessed using Spearman's correlation analysis in the TIMER3 database. Results are shown as lollipop plots representing Spearman correlation coefficients (*ρ*) between gene expression and specific immune cell types, with FDR corrections applied to adjust for multiple testing across immune cell subsets.

### 2.11 Enrichment Analysis

To understand the functional role of co‐expressed genes of LIG1 and MSTO1, we performed integrated bioinformatics analyses. Co‐expression data for each gene were collected from cBioPortal. Further functional enrichment and pathway analyses were carried out using the Metascape platform [19] to identify statistically significant Gene Ontology (GO) terms, evaluated using Benjamini‐Hochberg FDR adjustments and represented as −log10 (*p*) values. To search for possible functional protein associations, protein–protein interaction (PPI) networks were created for the identified co‐expressed genes. Functional sub‐clusters within the interactome were identified by MCODE (Molecular Complex Detection) to identify dense connections of modules within these networks.

### 2.12 Statistical Analysis

Statistical analyses of quantitative data were performed using tools provided by the UALCAN portal. Differences in mRNA expression (from TCGA data) and protein expression (from CPTAC data) between normal liver tissue and HCC samples were evaluated using Student's *t*‐test. For survival analysis, overall survival was assessed using Kaplan–Meier analysis with the log‐rank test, and overall prognostic value was assessed using multivariate Cox proportional hazards regression (lifelines v0.30.3, Python, Efron approximation). To test the statistical significance of differential methylation, we compared the mean *β*‐values between primary tumor tissues and normal liver tissues using an unpaired *t*‐test. ROC curve analysis was conducted using GraphPad Prism 10. To investigate the association between gene expression and immune cell infiltration, data were retrieved from the TIMER3 database, and lollipop plots were generated in GraphPad Prism 10. All *p*‐ or *q*‐values less than 0.05 were considered significant.

## 3 Results

### 3.1 Expression Analysis of Candidate Altered Genes in Hepatocellular Carcinoma (HCC) Versus Normal Tissues

**FIGURE 1:**
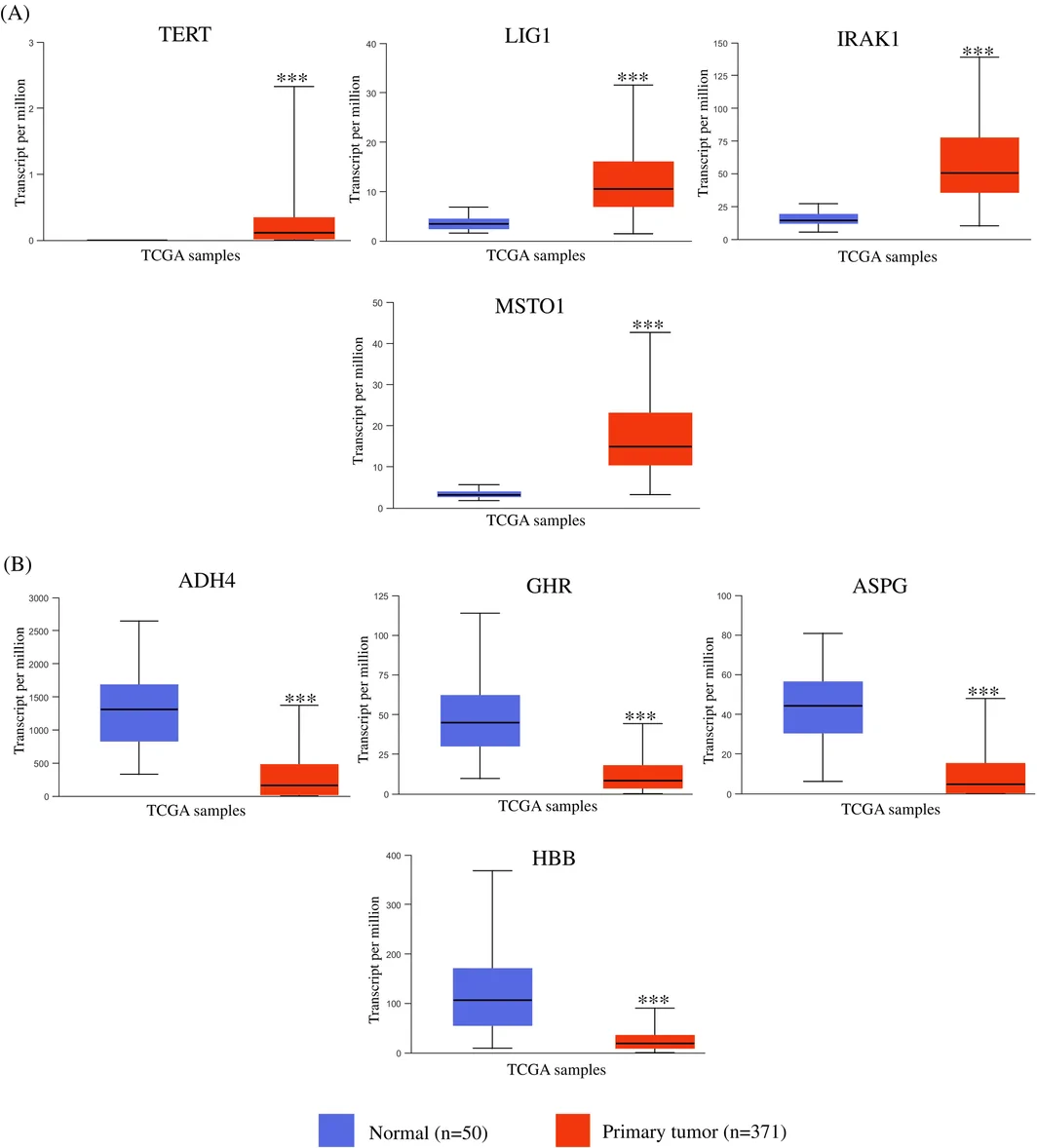
mRNA expression levels of the candidate overexpressed (A) and downregulated (B) genes in HCC (*n* = 371) compared with normal tissues (*n* = 50) based on TCGA data analyzed through the UALCAN portal (Student's *t*‐test). ***p < 0.001.

mRNA expression levels (transcripts per million) of the candidate DEGs, presented in Table 1, were examined in HCC using the UALCAN portal (TCGA‐LIHC dataset), based on RNA‐seq data from HCC samples (*n* = 371) and adjacent normal liver tissues (*n* = 50). Expression levels, visualized as box plots and including the median expression levels, revealed a statistically significant upregulation of TERT, LIG1, IRAK1, and MSTO1 transcripts (Figure 1A), whilst a statistically significant downregulation of ADH4, GHR, ASPG, and HBB transcripts in HCC tissues versus normal tissues (Figure 1B). Together, these findings suggest that these genes may be associated with HCC and may hold potential clinical relevance.

**TABLE 1:** Differential expression of the eight candidate genes (TCGA‐LIHC, HCC tumor *n* = 371 vs. adjacent normal liver *n* = 50).

Gene	Gene Name	log2FC	*p*	FDR (q)
TERT	Telomerase Reverse Transcriptase	+3.79	2.25e‐77	1.80e‐76
MSTO1	Misato Mitochondrial Distribution and Morphology Regulator 1	+1.72	9.16e‐60	3.66e‐59
ASPG	Asparaginase	−4.58	8.25e‐59	2.20e‐58
ADH4	Alcohol dehydrogenase 4	−4.37	4.61e‐58	9.23e‐58
GHR	Growth hormone receptor	−3.03	4.41e‐39	7.06e‐39
LIG1	DNA Ligase 1	+1.12	4.55e‐26	6.07e‐26
IRAK1	Interleukin 1 Receptor Associated Kinase 1	+1.25	4.00e‐25	4.58e‐25
HBB	Hemoglobin Subunit Beta	−2.78	2.45e‐22	2.45e‐22

*Note:* log2 fold‐change (tumor vs. normal, log2‐transformed RNA‐seq V2 RSEM expression) and unpaired Welch's *t*‐test, *p*‐values, with Benjamini‐Hochberg FDR correction applied across the eight genes tested.

To complement these UALCAN‐based comparisons with directly reproducible estimates of effect size and significance, we independently computed log2 fold change and Benjamini‐Hochberg FDR‐adjusted significance for all eight candidate genes using the same TCGA‐LIHC RNA‐seq dataset (tumor *n* = 371, normal *n* = 50; Table 1). All eight genes showed substantial and highly significant dysregulation (|log2FC| range 1.12–4.58; FDR *q*‐values from 2.4 × 10^−22^ to 1.8 × 10^−76^), confirming a balanced panel of four significantly upregulated genes (TERT: log2FC = +3.79; MSTO1: +1.72; IRAK1: +1.25; LIG1: +1.12) and four significantly downregulated genes (ASPG: log2FC = −4.58; ADH4: −4.37; GHR: −3.03; HBB: −2.78).

### 3.2 Association Between Candidate Genes and Tumor Grades and Stages

**FIGURE 2:**
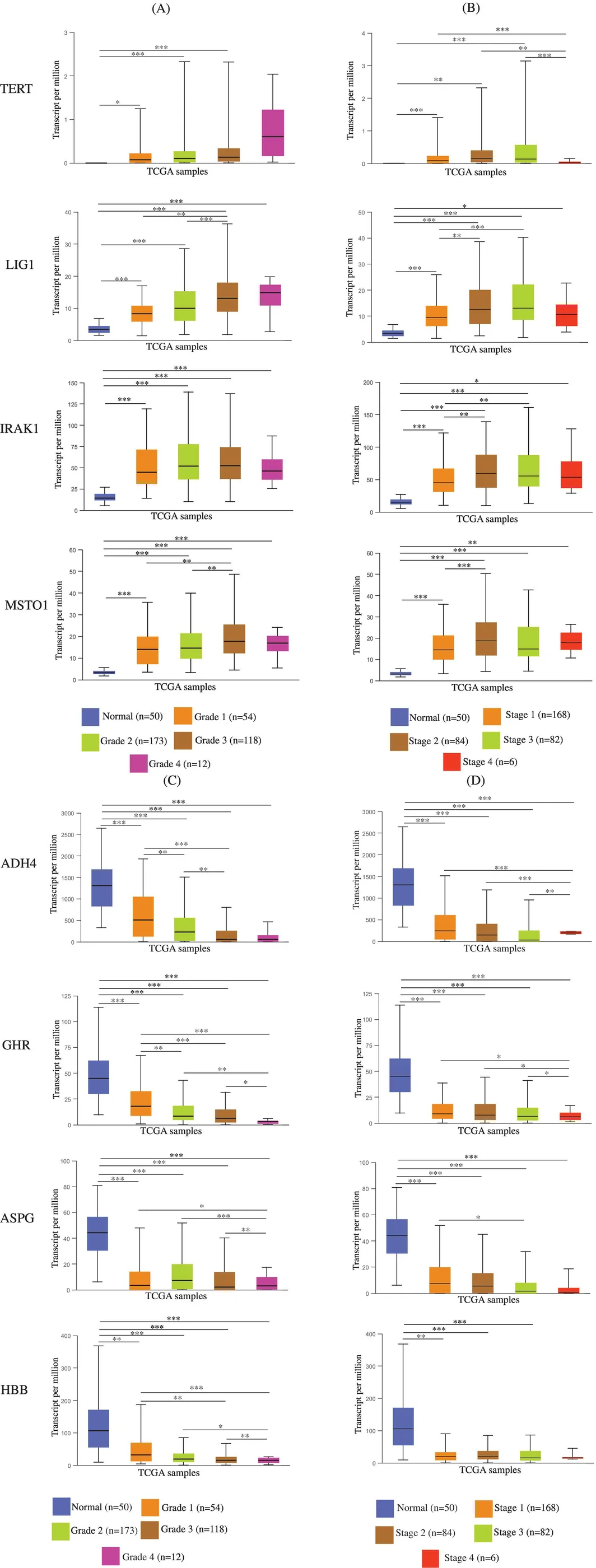
Expression levels of the candidate overexpressed genes in HCC according to tumor grades (A) and tumor stages (B), and expression levels of the downregulated candidate genes in HCC according to tumor grades (C) and tumor stages (D) (Student's *t*‐test). Statistical significance is indicated as follows: *p < 0.05; **p < 0.01; ***p < 0.001.

We next evaluated the association between mRNA expression of the candidate genes and clinico‐pathological features of HCC patients using the UALCAN web portal. Tumor grades were classified as Grade 1 (well differentiated, low grade, *n* = 54), Grade 2 (moderately differentiated, intermediate grade, *n* = 173), Grade 3 (poorly differentiated, high grade, *n* = 118), and Grade 4 (undifferentiated, high grade, *n* = 12), whilst tumor stages included Stage 1 (*n* = 168), Stage 2 (*n* = 84), Stage 3 (*n* = 82), and Stage 4 (*n* = 6). As shown in Figure 2A, expression levels of the upregulated genes were significantly correlated with tumor grade, with higher expression observed in advanced grades. A similar pattern was seen across tumor stages, where patients in later stages exhibited markedly elevated expression (Figure 2B), indicating a potential role in driving HCC progression.

Similarly, analysis of the downregulated genes (ADH4, GHR, ASPG, and HBB) revealed significant association with tumor grades and stages. As shown in Figure 2C, their mRNA expression level progressively decreased with increasing tumor grade, whilst patients with more advanced stages exhibited markedly lower expression (Figure 2D), suggesting that the downregulation of these genes may contribute to HCC progression.

### 3.3 Protein Expression Levels of Candidate Genes in Normal and HCC Tissues

**FIGURE 3:**
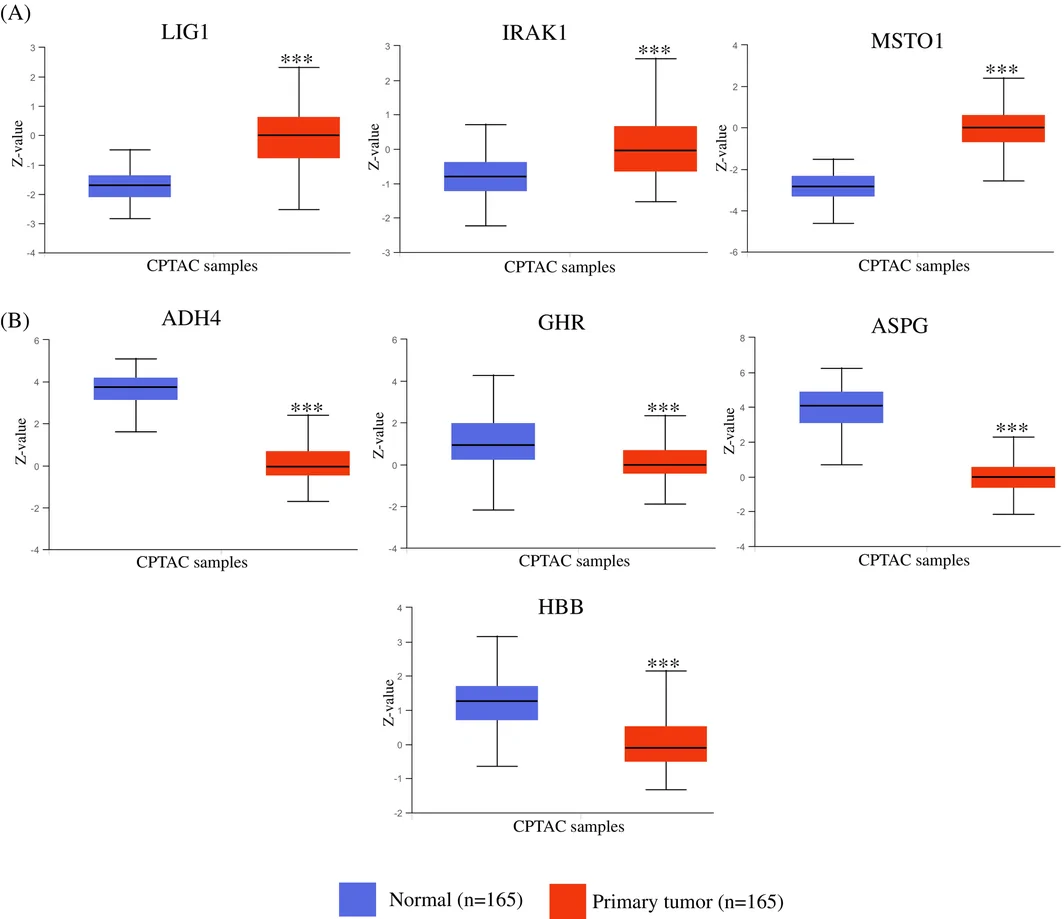
Protein expression levels of overexpressed (A) and downregulated (B) candidate genes in HCC (*n* = 165) and normal tissues (*n* = 165) based on the CPTAC database via the UALCAN web portal (Student's *t*‐test). ***p < 0.001.

To extend our transcript‐level findings, we examined protein expression levels of the candidate genes using CPTAC data via the UALCAN portal. Expression levels were evaluated in HCC samples (*n* = 165) and matched normal liver tissues (*n* = 165), and the median values are presented in Figure 3. As shown in Figure 3A, protein levels of LIG1, IRAK1, and MSTO1 were significantly elevated in HCC, aligning with their mRNA overexpression. On the other hand, ADH4, GHR, ASPG, and HBB protein expression levels were significantly reduced in HCC (Figure 3B), consistent with transcriptomic data. Proteomic data for TERT were unavailable, potentially due to limitations in mass spectrometry sensitivity, post‐transcriptional regulation, or the exclusion of low‐abundance or poorly quantified proteins. These findings further support the clinical relevance of the examined genes in HCC.

### 3.4 Immunohistochemistry Protein Expression Level of Our Candidate Genes in Normal and HCC Biopsies

**FIGURE 4:**
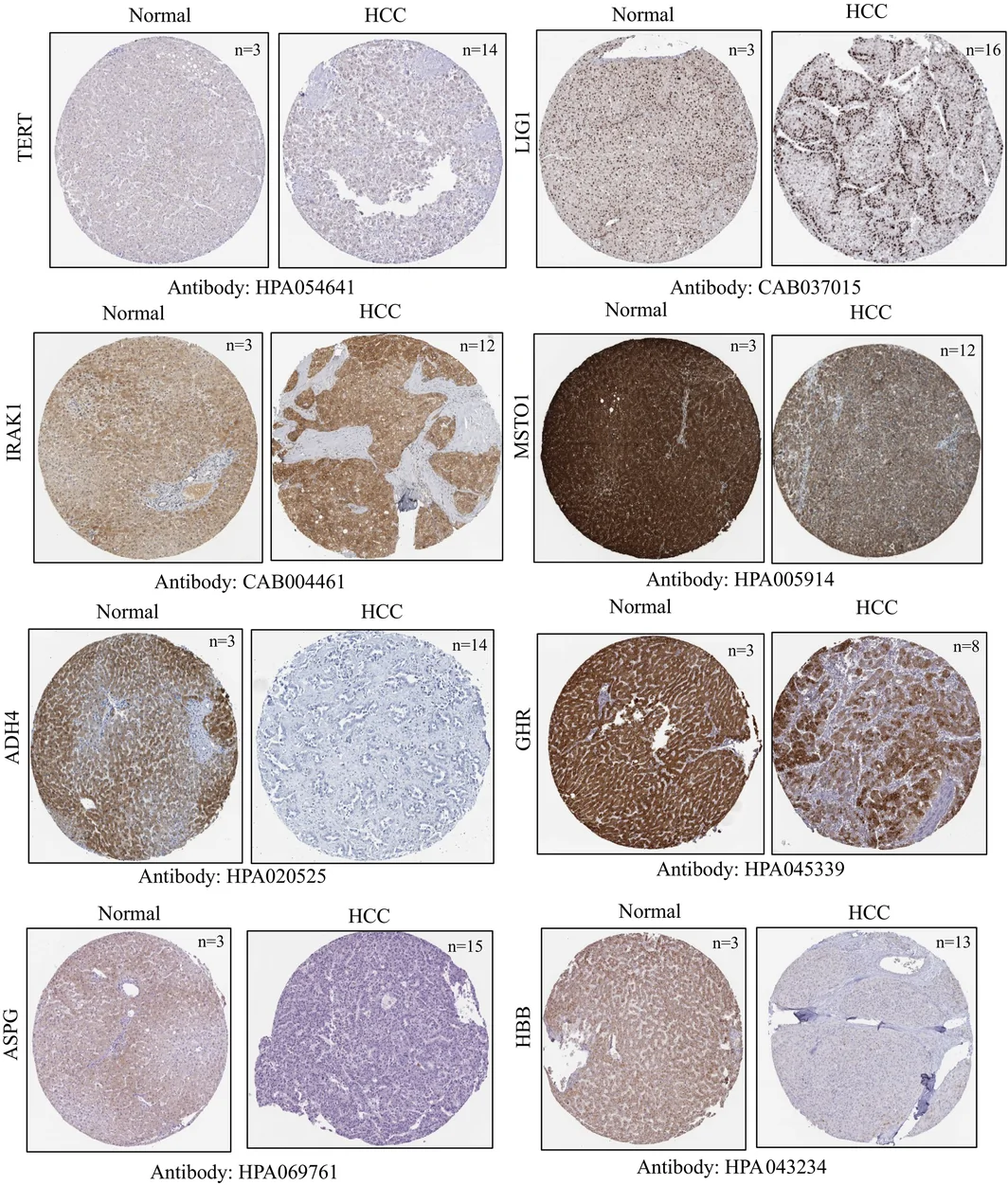
Protein expression levels of candidate genes in normal and HCC biopsies assessed by immunohistochemistry (Data from the human protein Atlas).

Immunohistochemical analysis in HPA revealed comparable protein levels of TERT and MSTO1 in both normal and HCC liver tissues. TERT staining was weak or absent in fewer than 25% of tumor cells, whereas MSTO1 was strongly expressed in both tissue types. Elevated protein levels of LIG1 and IRAK1 in HCC confirmed prior findings. ADH4 was highly expressed in normal hepatocytes but markedly downregulated or absent in HCC, with similar downregulation observed for HBB. ASPG showed weak expression in normal tissue but was undetectable in HCC, while GHR maintained moderate to high expression in both normal and cancerous tissues (Figure 4).

It is important to note that HPA's immunohistochemistry is based on a limited number of representative samples, restricting statistical analysis and the detection of subtle or variable expression patterns, thereby limiting the robustness of quantitative conclusions.

### 3.5 Diagnostic Potential and Predictive Accuracy of Candidate Genes in HCC

**FIGURE 5:**
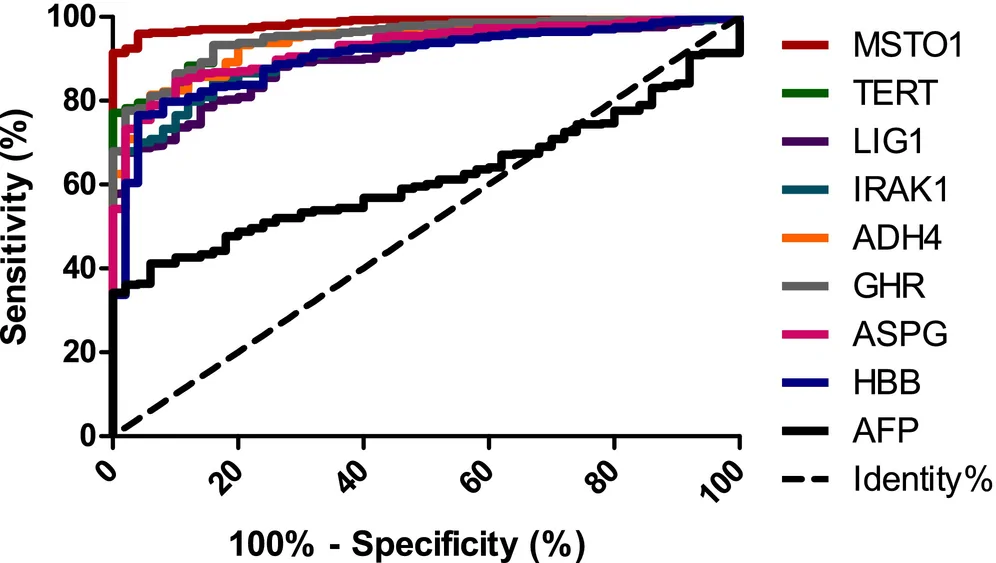
Receiver operating characteristic (ROC) curve to evaluate the diagnostic performance of candidate genes. The curves show the sensitivity and specificity of each gene to discriminate HCC (*n* = 371) from normal liver tissue (*n* = 50). The dashed diagonal line is the identity line (AUC = 0.5).

To determine the diagnostic accuracy of the candidate genes, ROC analysis was conducted to differentiate HCC tissue samples (*n* = 371) from normal liver tissues (*n* = 50). Area under the curve (AUC) was used as the main parameter for evaluation of sensitivity and specificity of each candidate gene (Table 2 and Figure 5). Results showed that most of the identified candidates had significant diagnostic accuracy, with AUC scores above 0.89. Among the studied genes, the best diagnostic potential was observed for MSTO1 (AUC = 0.9873; 95% CI: 0.9791–0.9955; *p* < 0.0001), followed by GHR (AUC = 0.9514) and ADH4 (AUC = 0.9421). All of the analyzed genes, except AFP (AUC = 0.6165), exhibited high diagnostic efficiency (*p* < 0.0001) for distinguishing cancerous from healthy tissue. In particular, it is necessary to emphasize that all candidate genes identified in this study showed greater diagnostic efficiency than the AFP gene, which is used as a conventional HCC biomarker. It should be noted that this AFP AUC (0.6165) reflects tumor‐versus‐normal AFP mRNA expression from TCGA‐LIHC, evaluated here on the same footing as the other candidate transcripts; it is therefore not directly equivalent to the diagnostic performance of the serum AFP protein assay used in clinical practice, which is generally reported with higher discriminative accuracy (e.g., pooled AUC ~0.87 across case–control studies). The comparison above should accordingly be interpreted as a like‐for‐like transcriptomic benchmark rather than a head‐to‐head comparison with the clinical AFP test.

**TABLE 2:** Diagnostic accuracy parameters of candidate genes in patients with HCC (*n* = 371) versus normal controls (*n* = 50).

Genes	AUC	Standard error	*p*	95% Confidence interval	
				Lower bound	Upper bound
MSTO1	0.9873	0.004175	< 0.0001	0.9791	0.9955
TERT	0.9390	0.01201	< 0.0001	0.9154	0.9625
LIG1	0.8965	0.01778	< 0.0001	0.8617	0.9314
IRAK1	0.9119	0.01591	< 0.0001	0.8807	0.9431
ADH4	0.9421	0.01336	< 0.0001	0.9159	0.9683
GHR	0.9514	0.01168	< 0.0001	0.9285	0.9743
ASPG	0.9243	0.01501	< 0.0001	0.8949	0.9537
HBB	0.9030	0.01883	< 0.0001	0.8661	0.9399
AFP	0.6165	0.02972	0.007457	0.5583	0.6748

### 3.6 Prognostic Value of Our Candidate Genes

**FIGURE 6:**
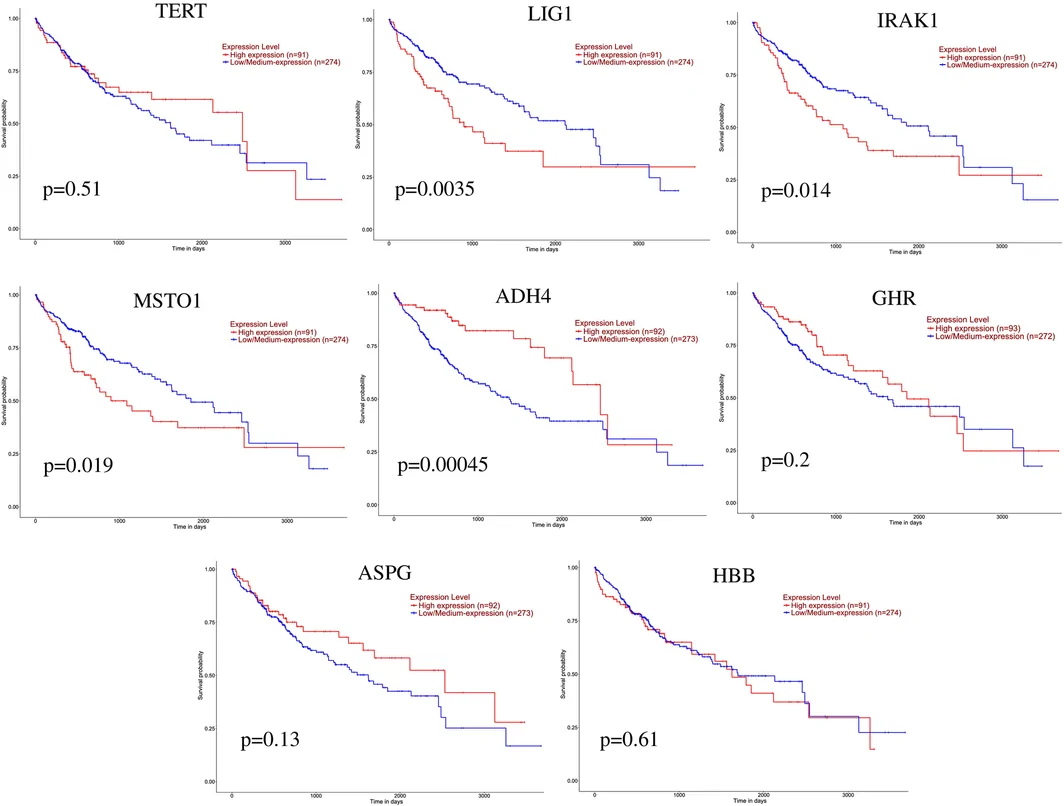
Survival probability based on high and low/medium expression of our candidate overexpressed and downregulated genes in HCC patients (*n* = 365) (log‐rank test). Results were considered significant for *p*‐values < 0.05.

To assess the prognostic value of the selected genes in HCC, we analyzed survival data using Kaplan–Meier survival plots based on TCGA clinical and gene expression data. As shown in Figure 6, high expression of LIG1, IRAK1, and MSTO1 was significantly associated with reduced survival probability (*p* = 0.0035, 0.014, and 0.019, respectively). Among the downregulated genes, low ADH4 expression was also linked to poorer survival (*p* = 0.00045). These findings suggest that LIG1, IRAK1, MSTO1, and ADH4 may serve as prognostic candidate biomarkers for predicting the survival of HCC patients. On the other hand, multivariate Cox regression adjusting for clinic‐pathological covariates showed that only MSTO1 remained significant after adjustment for stage, grade, age, sex, and aetiology, whilst none remained significant after additional adjustment for AFP status.

### 3.7 Cox Multivariate Analysis

**FIGURE 7:**
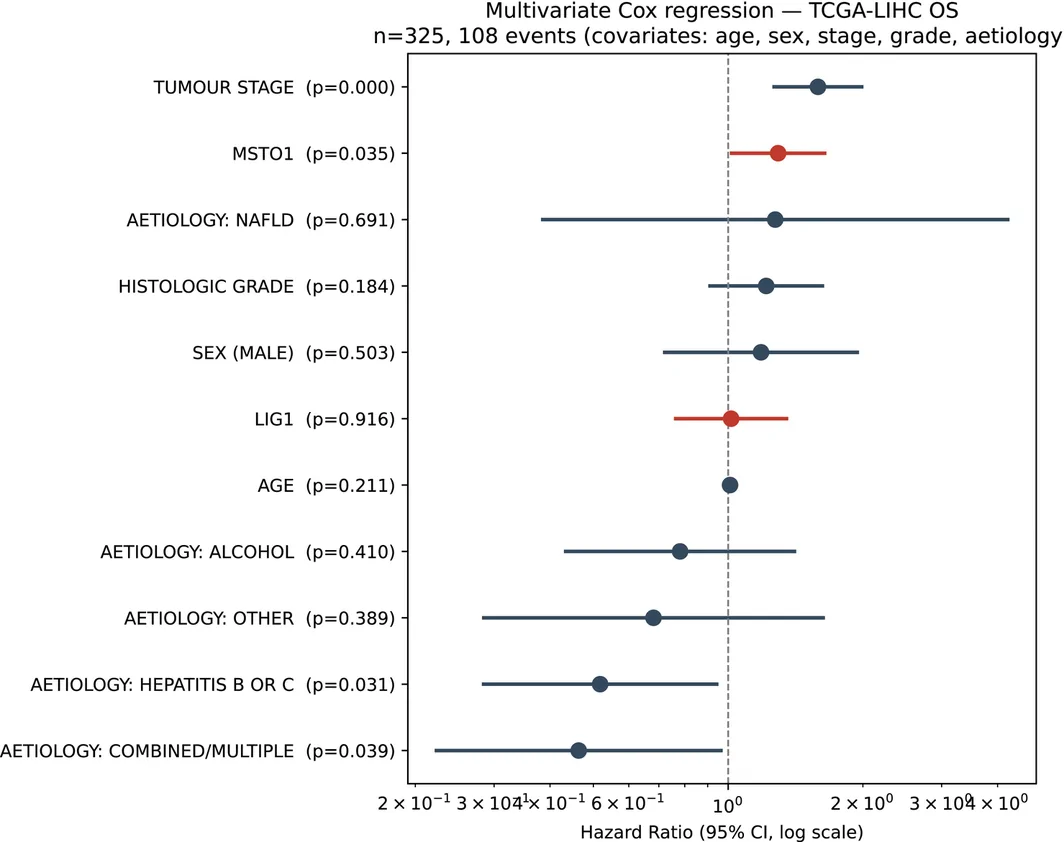
Multivariate Cox proportional hazards regression. Model 1 (overall survival, TCGA‐LIHC; *n* = 325, 108 events; covariates: MSTO1 and LIG1 expression, age, sex, tumor stage, histologic grade, etiology). Forest plot shows hazard ratios with 95% confidence intervals on a log scale (dashed line = HR 1); MSTO1 and LIG1 are highlighted in red. Etiology categories are coded relative to a reference category of no history of primary risk factors. The “hepatitis B or C” category combines hepatitis B (*n* = 75) and hepatitis C (*n* = 32) cases, and “combined/multiple” denotes patients with more than one recorded risk factor (predominantly alcohol with hepatitis B or C).

To determine whether MSTO1 and LIG1 are independently prognostic, multivariate Cox proportional hazards regression was performed (Figure 7). In Model 1 (adjusted for age, sex, tumor stage, grade and etiology; *n* = 325, 108 events, Table 3), MSTO1 expression (HR = 1.29, 95% CI: 1.02–1.64, *p* = 0.035) and tumor stage (HR = 1.59, *p* < 0.001) were independent predictors of overall survival, with tumor stage exerting the strongest effect. LIG1 expression showed no significant association with survival (HR = 1.02, 95% CI: 0.76–1.35, *p* = 0.916). In Model 2 (Table 4), with additional adjustment for serum AFP status (*n* = 254, 75 events), MSTO1 was no longer a significant independent predictor of overall survival (HR = 1.12, 95% CI: 0.83–1.50, *p* = 0.458). Instead, tumor stage (HR = 1.46, *p* = 0.003) and histologic grade (HR = 1.55, *p* = 0.016) were identified as the dominant independent predictors. Treatment/therapy status was recorded for only 101/371 patients (27%) in this cohort and could not be reliably incorporated into the multivariate model without severely reducing statistical power. These results indicate that MSTO1 expression was associated with overall survival in a multivariate Cox proportional hazards model adjusted for clinicopathologic covariates, but not after further adjustment for AFP status. Accordingly, we describe it as a candidate rather than a confirmed independent prognostic marker. LIG1 was not supported as an independent prognostic marker by either model.

**TABLE 3:** Multivariate Cox proportional hazards regression, Model 1 (overall survival, TCGA‐LIHC; *n* = 325, 108 events; adjusted for age, sex, tumor stage, grade and aetiology).

Covariate	HR	95% CI	*p*
MSTO1 expression (log2)	1.29	1.02–1.64	0.035*
LIG1 expression (log2)	1.02	0.76–1.35	0.916
Tumor stage (I–IV)	1.59	1.27–1.99	< 0.001*
Histologic grade (G1–G4)	1.22	0.91–1.62	0.184
Age (years)	1.01	0.99–1.03	0.211
Sex (male vs. female)	1.18	0.72–1.94	0.503

* (*p* < 0.05).

**TABLE 4:** Multivariate Cox proportional hazards regression, Model 2 (overall survival, TCGA‐LIHC; *n* = 254, 75 events; additionally adjusted for serum AFP status).

Covariate	HR	95% CI	*p*
MSTO1 expression (log2)	1.12	0.83–1.50	0.458
LIG1 expression (log2)	0.85	0.58–1.25	0.414
Tumor stage	1.46	1.14–1.89	0.003*
Histologic grade	1.55	1.08–2.22	0.016*
AFP status (elevated)	1.22	0.71–2.08	0.474
Age (years)	1.03	1.01–1.05	0.005*
Sex (male vs. female)	0.83	0.51–1.38	0.476

* (*p* < 0.05).

Because eight candidate genes were tested for a survival association, Benjamini‐Hochberg FDR correction was applied to the corresponding log‐rank *p*‐values (Table 5). All four genes significant at the nominal *p* < 0.05 threshold (ADH4, *p* = 0.00045; LIG1, *p* = 0.0035; IRAK1, *p* = 0.014; MSTO1, *p* = 0.019) remained significant after correction (*q* = 0.0036, 0.014, 0.037 and 0.038, respectively), while the four non‐significant genes (GHR, ASPG, TERT, HBB) remained non‐significant, indicating that the survival associations reported here are robust to multiple‐testing correction.

**TABLE 5:** Benjamini‐Hochberg FDR correction of gene‐level overall‐survival log‐rank *p*‐values (family of eight candidate genes).

Gene	Nominal *p*	BH‐adjusted *q*	Significant (*q* < 0.05)
ADH4	0.00045	0.0036	Yes
LIG1	0.0035	0.014	Yes
IRAK1	0.014	0.037	Yes
MSTO1	0.019	0.038	Yes
GHR	0.13	0.208	No
ASPG	0.2	0.267	No
TERT	0.51	0.583	No
HBB	0.61	0.61	No

Given the etiological heterogeneity of HCC, exploratory log‐rank tests compared high versus low MSTO1 expression (etiology‐specific median split) within each etiology category available in our dataset (no history of primary risk factors; hepatitis B or C; alcohol consumption; combined/multiple risk factors, predominantly alcohol with hepatitis B or C; non‐alcoholic fatty liver disease; other) (Table 6). MSTO1's prognostic association was not uniform: it was significant among patients with alcohol‐related HCC (*n* = 68, *p* = 0.0071), but not significant among patients with hepatitis B‐ or C‐related HCC (*n* = 107, *p* = 0.067), no history of primary risk factors (*n* = 91, *p* = 0.232), or combined/multiple risk factor exposure (*n* = 58, *p* = 0.165); the non‐alcoholic fatty liver disease and other/unspecified categories combined (*n* = 27) were too small for reliable subgroup analyses. This indicates that MSTO1's prognostic value may be etiology‐dependent, and appears strongest in alcohol‐related HCC, warranting validation within specific etiological subgroups rather than being assumed uniform across HCC.

**TABLE 6:** Exploratory MSTO1 aetiology‐subgroup survival analysis (high vs. low MSTO1 expression, aetiology‐specific median split; log‐rank test).

Aetiology category	*n*	MSTO1 high vs. low log‐rank *p*	Interpretation
No history of primary risk factors	91	0.232	Not significant
Hepatitis B or C	107	0.067	Borderline
Alcohol consumption	68	0.0071	Significant
Combined/multiple risk factors (predominantly alcohol with hepatitis B or C)	58	0.165	Not significant
Non‐alcoholic fatty liver disease | Other (combined)	27	Not tested	Sample too small

### 3.8 Evaluation of Knock‐Out (CRISPR) of Our Candidate Genes on HCC

Gene dependency analysis using DepMap gene effect scores across 23 HCC cell lines revealed variable impacts on cell viability. Each score reflects the importance of a gene for the survival of a specific cell line, with more negative values indicating greater dependency. Scores below −0.5 denote strong dependency, whilst values near zero suggest non‐essentiality.

**FIGURE 8:**
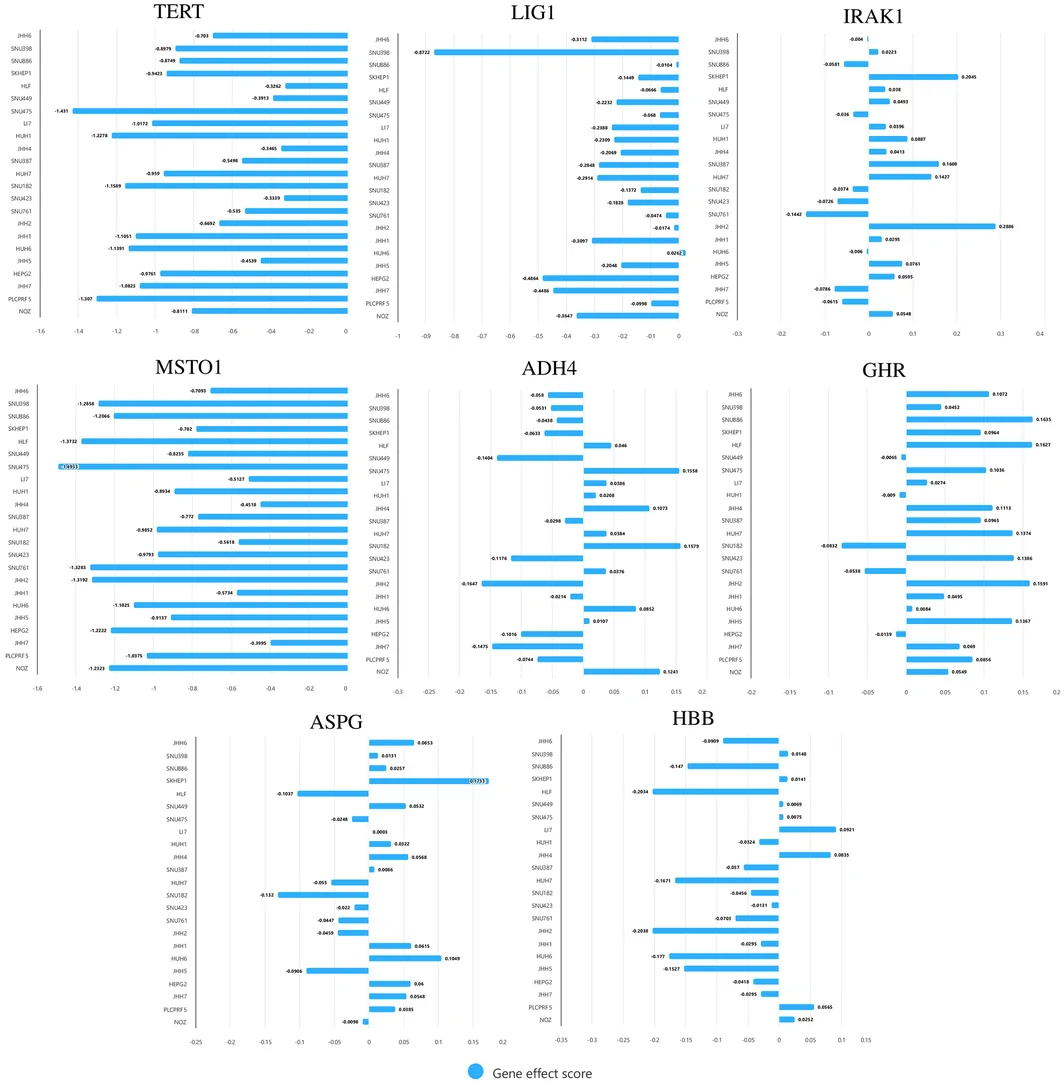
Gene effect scores of candidate genes in liver cancer cell lines based on DepMap analysis. Negative scores imply cell growth inhibition and/or death following gene knockout. Scores are normalized such that non‐essential genes have a median score of 0, and more negative values indicate stronger dependency. A median score of −0.5 is used to define essential genes, with scores around −1 representing core essential genes whose loss severely impairs cell survival.

TERT and MSTO1 demonstrated strong dependency, with consistently negative scores below −0.5 across nearly all HCC cell lines, highlighting their essential roles in HCC cell survival. LIG1 exhibited moderate dependency in several cell lines. In contrast, IRAK1, ADH4, GHR, ASPG, and HBB generally had scores near zero or positive, indicating non‐essential roles in HCC viability (Figure 8).

These findings identify TERT and MSTO1 as critical dependency genes in HCC, while LIG1 exhibits moderate importance. Collectively, these genes may represent potential therapeutic dependencies or candidate vulnerabilities in HCC, although further experimental validation is required to confirm their functional and clinical relevance.

### 3.9 Pan‐Cancer Expression Profiling Identifies Candidate Genes With Upregulated Expression in HCC

**FIGURE 9:**
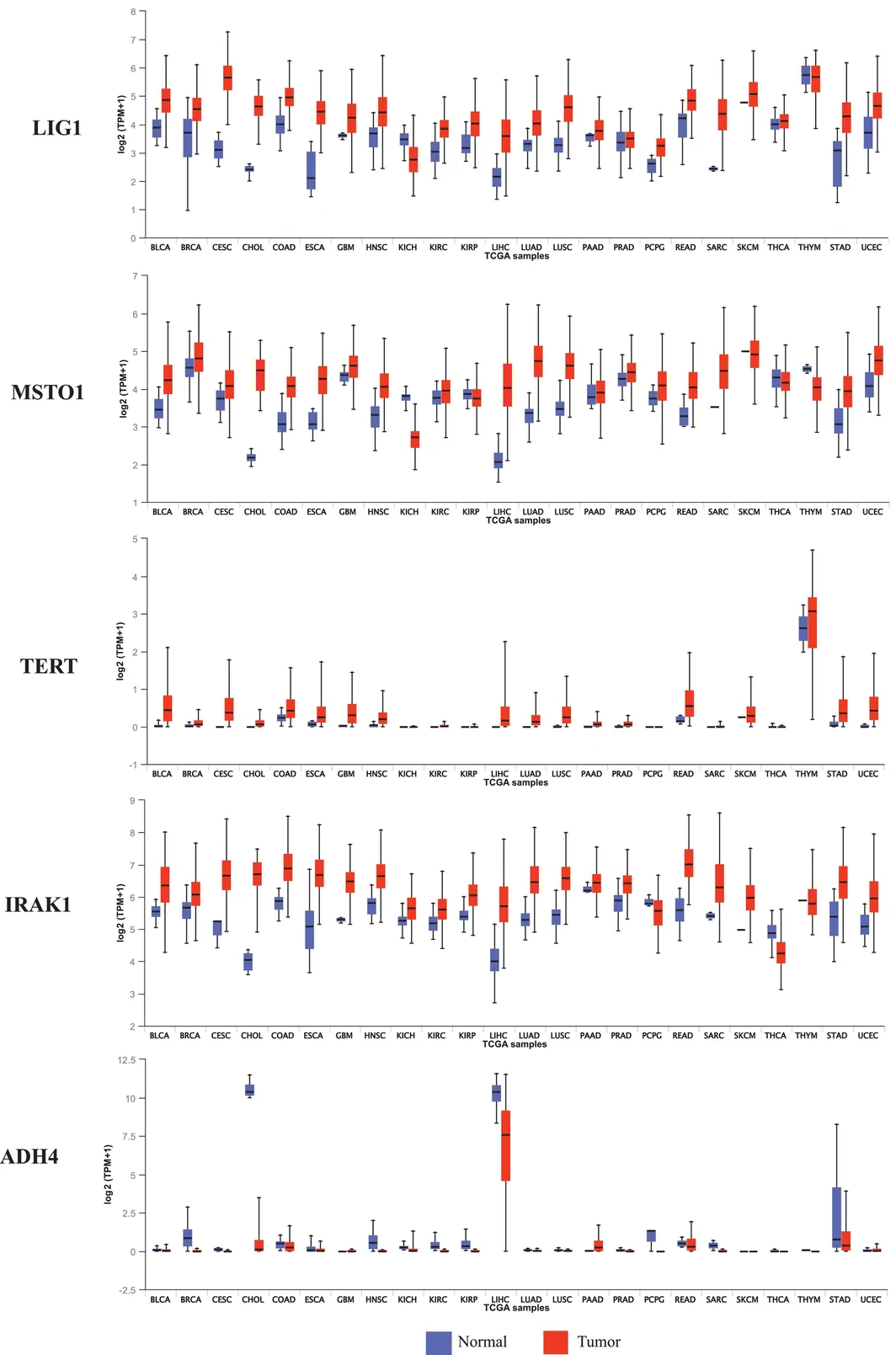
Pan‐cancer differential expression of candidate genes in tumor versus normal tissues across TCGA datasets. Boxplots illustrate log2(TPM + 1) expression levels of LIG1, MSTO1, TERT, IRAK1, and ADH4 across multiple cancer types. Red boxes represent tumor samples, and blue boxes represent matched normal tissues. Data were obtained via the UALCAN portal using TCGA transcriptomic datasets. BLCA, Bladder urothelial carcinoma; BRCA, Breast invasive carcinoma; CESC, Cervical squamous cell carcinoma; CHOL, Cholangiocarcinoma; COAD, Colon adenocarcinoma; ESCA, Esophageal carcinoma; GBM, Glioblastoma multiforme; HNSC, Head and neck squamous cell carcinoma; KICH, Kidney chromophobe; KIRC, Kidney renal clear cell carcinoma; KIRP, Kidney renal papillary cell carcinoma; LIHC, Liver hepatocellular carcinoma; LUAD, Lung adenocarcinoma; LUSC, Lung squamous cell carcinoma; PAAD, Pancreatic adenocarcinoma; PCPG, Pheochromocytoma and paraganglioma; PRAD, Prostate adenocarcinoma; READ, Rectal adenocarcinoma; SARC, Sarcoma; SKCM, Skin cutaneous melanoma; STAD, Stomach adenocarcinoma; THCA, Thyroid carcinoma; THYM, Thymoma; UCEC, Uterine corpus endometrial carcinoma.

To assess the cancer‐type specificity of our candidate genes, we focused on those showing significant relevance to HCC based on their association with patient survival or HCC cell line viability. Consequently, TERT, LIG1, IRAK1, MSTO1, and ADH4 were selected for further analysis, as they demonstrated consistent expression patterns and potential functional importance in HCC (Figure 9).

We examined the transcript levels of our candidate genes across tumor and matched normal tissues using the TCGA pan‐cancer dataset. LIG1, MSTO1, TERT, and IRAK1 were significantly upregulated in multiple cancer types, including LIHC. While LIG1, MSTO1, and IRAK1 showed broad overexpression across various malignancies, TERT exhibited a more restricted pattern, with marked upregulation in LIHC and limited expression in normal tissues. In contrast, ADH4 was notably downregulated in LIHC whilst similar trends were observed in cholangiocarcinoma (CHOL) and stomach adenocarcinoma (STAD), indicating a more liver‐specific expression profile. Collectively, these results highlight that while LIG1, MSTO1, TERT, and IRAK1 likely participate in general oncogenic processes shared across malignancies, ADH4 stands out as a liver‐specific candidate whose dysregulation may reflect its unique metabolic function in hepatocytes, reinforcing its particular relevance to HCC biology.

### 3.10 Genomic Alterations in Selected Genes Across HCC Cohorts

**FIGURE 10:**
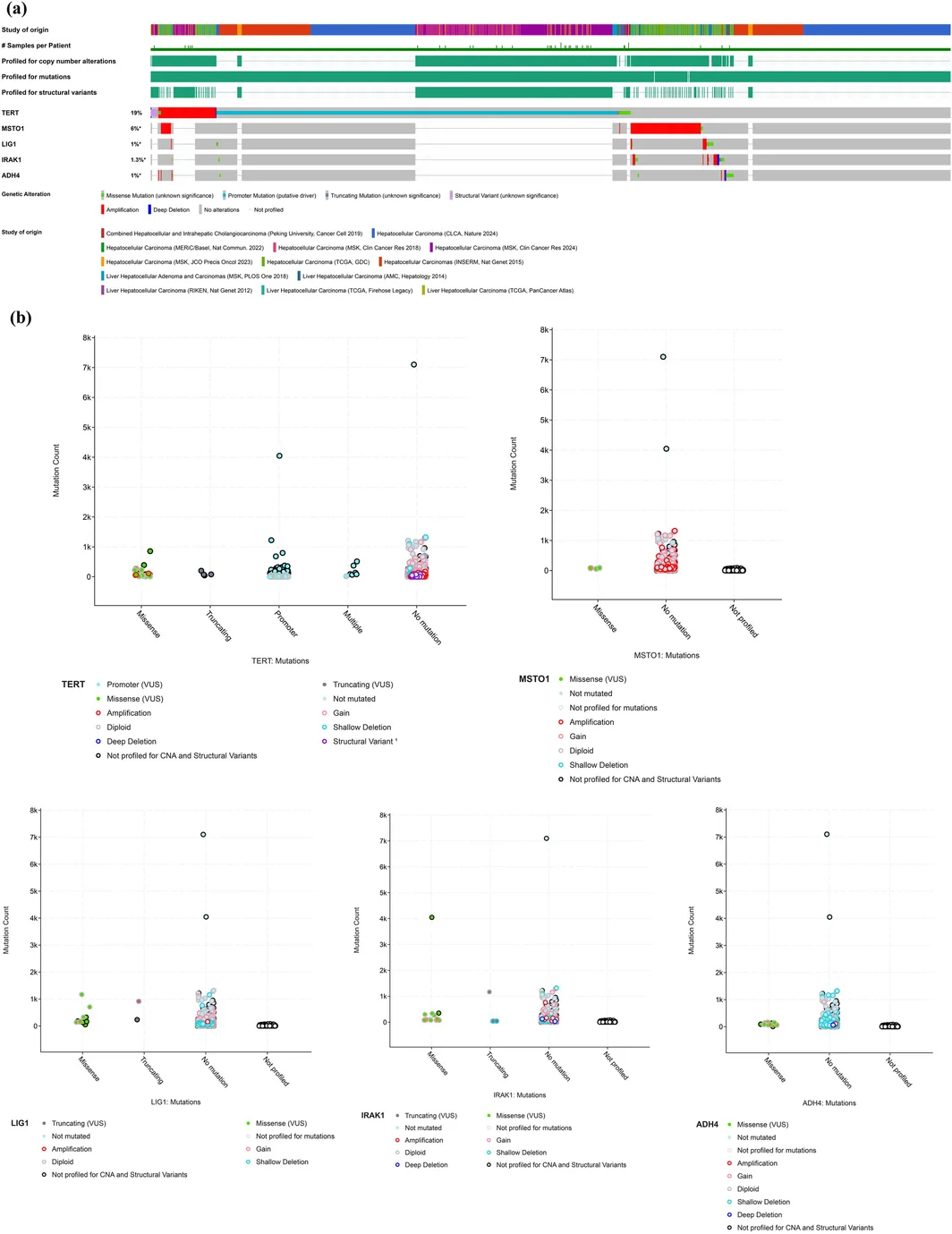
Genomic alterations in TERT, MSTO1, LIG1, IRAK1, and ADH4 across hepatocellular carcinoma cohorts. (a) Frequency and types of genetic alterations observed in five selected genes across 4026 samples from 13 liver cancer studies. Alteration types include promoter mutations, amplifications, truncating mutations, structural variants, and missense mutations of unknown significance. (b) Distribution of genomic alterations by types of mutations, indicating the heterogeneity and prevalence of specific gene alterations across hepatocellular carcinoma (HCC) datasets.

Analysis of genomic alterations across 4026 HCC samples revealed distinct mutation patterns among the five studied genes (TERT, MSTO1, LIG1, IRAK1, and ADH4). TERT exhibited the highest alteration frequency (19%), predominantly due to promoter mutations and amplifications, confirming its recurrent genomic dysregulation in HCC. MSTO1 showed a high alteration frequency (6%), mainly involving gene amplifications. In contrast, LIG1, IRAK1, and ADH4 demonstrated minimal mutation occurrences (1%), indicating overall genomic stability (Figure 10). Collectively, these findings highlight TERT as the most frequently altered gene in HCC whilst the other genes remain largely unmutated or diploid. Importantly, these results also indicate that the dysregulated expression observed for these genes in HCC is not necessarily driven by genomic mutations; instead, it may arise from transcriptomic, epigenetic, or post‐transcriptional mechanisms. Therefore, the absence of frequent mutations does not preclude their relevance as biomarkers, as gene expression alterations alone can still hold significant biological value in HCC.

### 3.11 Epigenetic Regulation of MSTO1 and ADH4 by Promoter Hypermethylation

**FIGURE 11:**
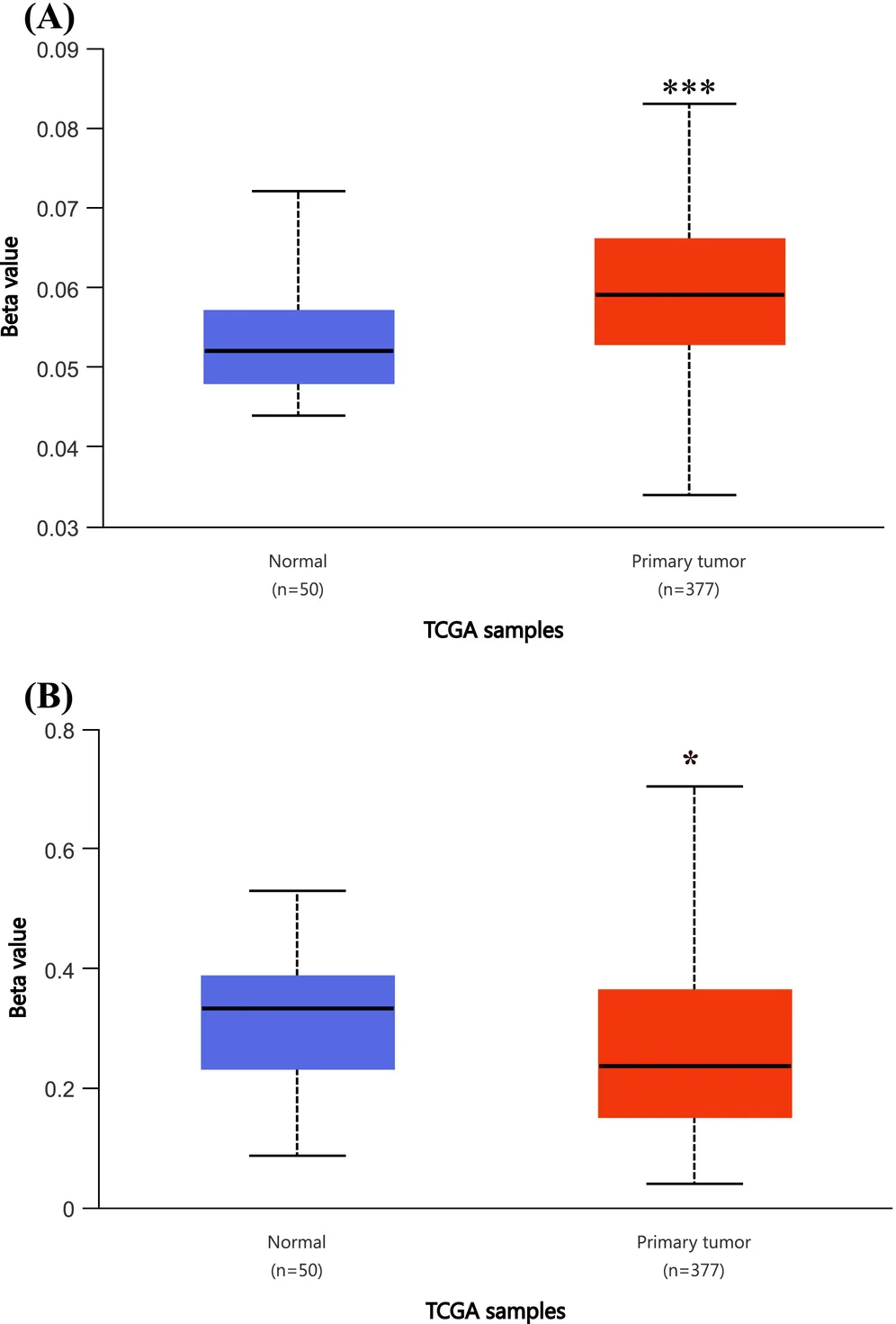
Promoter methylation levels of MSTO1 and ADH4 in HCC. (A) Analysis of MSTO1 promoter methylation in normal (*n* = 50) and primary tumor tissues (*n* = 377). (B) Analysis of ADH4 promoter methylation in normal (*n* = 50) and primary tumor tissues (*n* = 377), based on data from the UALCAN portal using TCGA samples (unpaired two‐sample *t*‐test). *p < 0.05; ***p < 0.001.

Our prior genomic alteration analysis of the HCC cohort showed that MSTO1 and ADH4 have low somatic mutation frequencies and are therefore unlikely to be primarily mutation‐driven. We therefore investigated whether promoter DNA methylation, a common epigenetic mechanism in HCC, underlies this dysregulated expression. To explore a possible epigenetic regulation of these candidate genes, we examined TCGA‐LIHC promoter methylation profiles using UALCAN (Figure 11). Our analysis showed that both genes were significantly differentially methylated in the promoter region of primary HCC tissues compared with normal liver tissues. The methylation level of the MSTO1 promoter was significantly higher in primary tumor samples (mean *β*‐value increase, *p* = 2.00 × 10^−5^), whilst the ADH4 promoter methylation was significantly lower in tumor tissues than in normal controls (*p* = 4.45 × 10^−2^). These data suggest a possible contribution of abnormal promoter methylation status to the dysregulation of gene expression of MSTO1 and ADH4 in HCC, providing evidence for epigenetic mechanisms underlying the observed gene expression patterns.

### 3.12 Correlation of MSTO1 and LIG1 Expression With Immune Cell Infiltration

**FIGURE 12:**
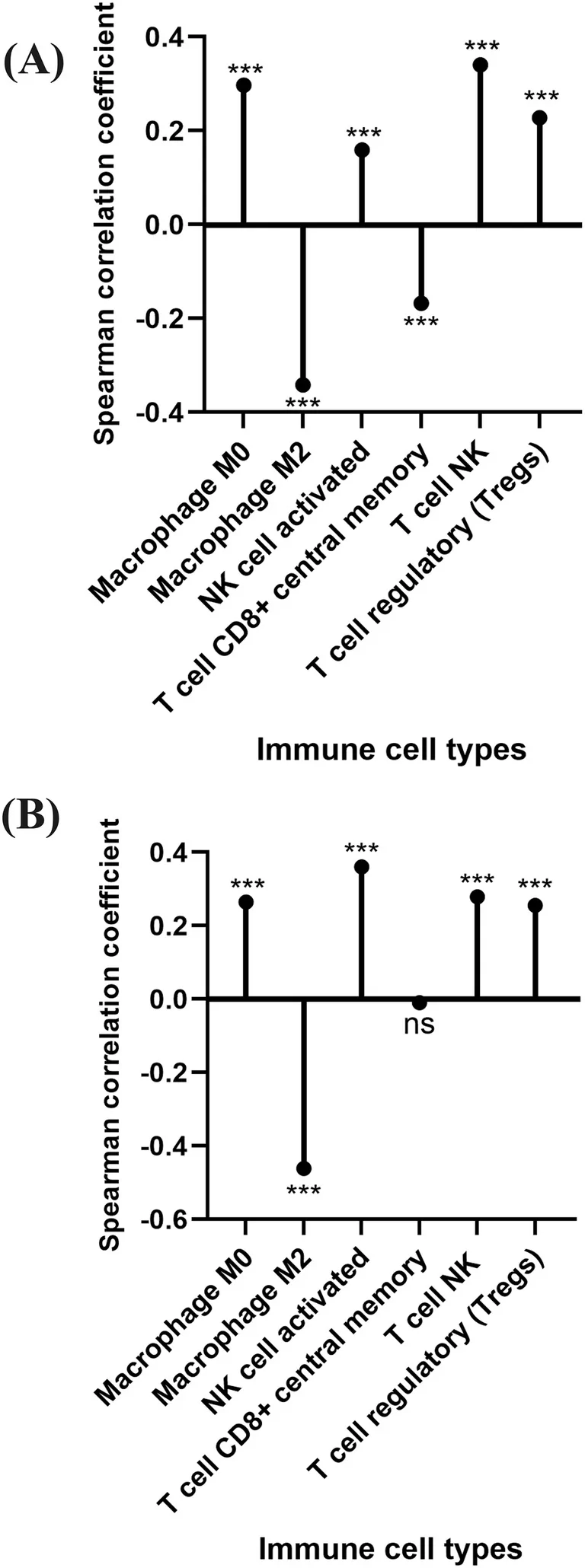
Spearman correlation of candidate gene expression and immune cell infiltration in HCC. (A) Lollipop plot of Spearman correlation coefficients (*ρ*) between MSTO1 expression and various immune cell types. (B) Lollipop plot of Spearman correlation coefficients (*ρ*) between LIG1 expression and types of immune cells. The vertical lines indicate the strength of the correlation, and asterisks show statistical significance (***p < 0.001; ns: Non‐significant).

To characterize the association of MSTO1 and LIG1 expression with the HCC tumor microenvironment, Spearman correlation analysis between their expression levels and the infiltration abundance of different immune cell types was performed. The correlation landscapes of MSTO1 (Figure 12A) and LIG1 (Figure 12B) showed different patterns of immune modulation. MSTO1 expression was significantly positively correlated with Macrophage M0, NK cell activated, T cell NK, and T cell regulatory (Tregs) populations and negatively correlated with Macrophage M2 cells. Furthermore, LIG1 exhibited a remarkable immune infiltration landscape where significant positive correlations were found between LIG1 expression and multiple cell subsets. These results suggest that MSTO1 and LIG1 may be important in the immune environment of HCC and may influence the recruitment and polarization of immune cells in the tumor microenvironment.

### 3.13 Biological Processes and Protein–Protein Interaction Modules of LIG1 and MSTO1 in HCC

**FIGURE 13:**
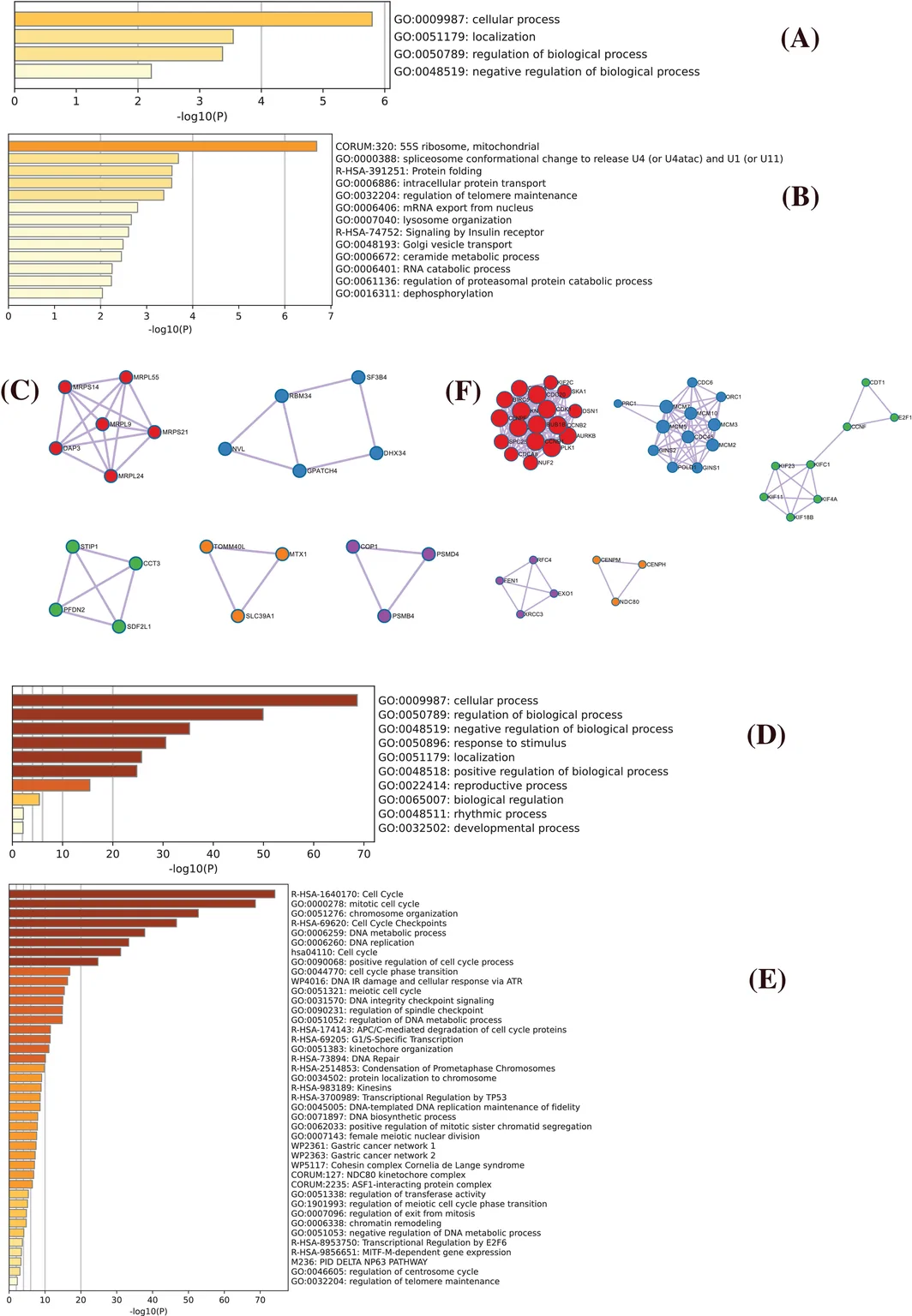
Functional enrichment and protein–protein interaction (PPI) network analysis of MSTO1 and LIG1 co‐expressed genes in HCC. (A) Summary of high‐level Gene Ontology (GO) biological processes for MSTO1‐correlated genes. (B) Detailed enriched functional pathways and molecular complexes for MSTO1‐correlated genes. (C) PPI network and MCODE functional modules for MSTO1. (D) Summary of high‐level GO biological processes for LIG1‐correlated genes. (E) Detailed enriched functional pathways for LIG1‐correlated genes. (F) PPI network and MCODE functional modules for LIG1.

Functional enrichment and protein–protein interaction (PPI) network analyses revealed distinct molecular landscapes for genes co‐expressed with LIG1 and MSTO1 (Figure 13). The genes co‐expressed with LIG1 were mainly involved in core cellular processes, including cell cycle, mitotic cell cycle regulation, DNA replication, and DNA damage response pathways (DNA IR damage and cellular response via ATR). Additional PPI network modularity analysis revealed robust clusters, such as cell cycle regulators and DNA repair components. On the other hand, the genes co‐expressed with MSTO1 were mainly involved in mitochondrial and translational functions, with PPI modules dominated by mitochondrial ribosomal and localization‐related proteins. Altogether, the obtained results indicate that LIG1 is an important component for chromosomal stability and cell proliferation, whilst the MSTO1 network co‐expression is strongly related to mitochondrial function and protein transport.

## 4 Discussion

Hepatocellular carcinoma (HCC) remains one of the most lethal malignancies worldwide, primarily due to its late diagnosis, high recurrence rate, and limited therapeutic options. In the present study, we aimed to identify potential diagnostic or prognostic biomarkers and therapeutic targets by analyzing DEGs in HCC using bioinformatics approaches. Specifically, we focused on a panel of 8 candidate genes (4 upregulated and 4 downregulated), evaluating their expression patterns, prognostic relevance, and potential vulnerability using publicly available datasets, including TCGA, CPTAC, Kaplan–Meier plots, DepMap, and HPA. Data from TCGA, CPTAC, Kaplan–Meier plots, and DepMap were analyzed using the integrative web resource UALCAN.

Among the upregulated genes, MSTO1 emerged as a novel and promising candidate biomarker in HCC. Our findings indicate that MSTO1 is significantly overexpressed at both the mRNA and protein levels in HCC tissues compared to normal liver tissue. Moreover, its expression levels correlate positively with tumor grades and stages, suggesting a potential role in HCC progression and underscoring its clinical significance.

Importantly, survival analysis revealed that high MSTO1 expression was associated with poor survival probability (*p* = 0.019), suggesting a potential prognostic role. This univariate association remained significant in multivariate Cox regression after adjustment for age, sex, tumor stage, grade, and etiology (HR = 1.29, *p* = 0.035), but did not persist after adding serum AFP status to the model (HR = 1.12, *p* = 0.458). Given that subgroup significance was limited to specific etiologic categories, MSTO1's prognostic value remains a hypothesis‐generating association requiring independent confirmation. Furthermore, functional dependency analysis revealed that MSTO1 is essential for the survival of most HCC cell lines, indicating its critical role in maintaining tumor cell viability.

Functionally, MSTO1 regulates mitochondrial fusion, a process critical for maintaining mitochondrial integrity and bioenergetics [20]. Its overexpression may promote elongated, stress‐resistant mitochondria, enhancing tumor cell survival, proliferation, and resistance to apoptosis. This shift in mitochondrial dynamics may also support immune evasion and a more aggressive tumor phenotype. Despite its biological importance, MSTO1 remains understudied in the context of HCC. Our findings suggest that it holds promise as both a diagnostic marker (as confirmed by ROC analysis) and a candidate therapeutic dependency in HCC. Furthermore, its prognostic value warrants confirmation across etiological subgroups and AFP‐adjusted models to better define its mechanistic role in tumor progression.

In contrast to MSTO1, TERT is a well‐established oncogene with a critical role in HCC [21] and numerous other malignancies [22]. In our study, TERT was significantly overexpressed in HCC tissues, as indicated by UALCAN. TERT encodes the catalytic subunit of telomerase, a ribonucleoprotein enzyme responsible for maintaining chromosomal integrity by adding telomeric repeats (5′‐TTAGGG‐3′) [23]. Human telomerase is composed of TERT and its RNA template component (TERC), which guides telomere elongation [24]. While telomerase is active in progenitor and cancer cells, its activity is low or absent in most postnatal somatic cells, resulting in progressive telomere shortening and entry into senescence. Reactivation of telomerase in somatic cells is therefore a critical event in oncogenesis. Beyond its canonical telomere‐maintaining function, telomerase also modulates Wnt signaling, apoptosis resistance, and cellular aging [23].

Somatic mutations in the TERT promoter (TERTp) are among the most frequent genetic events in HCC, occurring in 40%–60% of cases [25]. These alterations, including point mutations, insertions, deletions, and viral DNA integrations, enhance TERT expression, sustain telomere length, and promote cellular immortalization [21].

Evidence regarding their prognostic relevance is mixed; some studies found no prognostic value of TERT promoter (TERTp) mutations [26], whereas others reported associations with shorter survival [27] or poor prognosis when combined with the rs2853669 variant [28]. Moreover, TERTp mutations detected in circulating tumor DNA have also been proposed as prognostic markers [29]. Although TERT expression was not associated with survival probability in our study, its essential role in telomere maintenance underscores its clinical relevance. DepMap analysis further revealed that HCC cells are highly dependent on TERT for viability, suggesting a potential therapeutic dependency. Extensive literature supports TERT as a therapeutic target in HCC; preclinical studies have demonstrated that HCC cells exhibit telomerase addiction, and direct inhibition of TERT using antisense oligonucleotides effectively suppresses proliferation, induces DNA damage, and reduces tumor growth in xenograft models [30]. Taken together, despite the limited prognostic value observed in our dataset, TERT remains a validated diagnostic biomarker and a compelling therapeutic target in HCC.

Beyond MSTO1 and TERT, other DEGs such as LIG1 and IRAK1 were also found to be overexpressed at both mRNA and protein levels, with high expression significantly associated with poorer survival probability (*p* = 0.0035 and *p* = 0.014, respectively). DepMap analysis further revealed that HCC cells are dependent on LIG1, but not on IRAK1, thereby highlighting LIG1 as a potential therapeutic dependency. IRAK1, a kinase involved in TLR/IL‐1R‐mediated NF‐κB and MAPK signaling, is known to promote proliferation, inflammation, and apoptosis resistance in cancer [31]. Although IRAK1 inhibition has shown antitumor effects in HCC models [32], its lack of essentiality in DepMap likely reflects its immune‐modulatory role, which is not captured in vitro. By acting through TLR/IL‐1R–NF‐κB pathways, IRAK1 exerts its effects primarily within the tumor microenvironment rather than through direct effects on intrinsic tumor cell viability. Consistent with this, IRAK1 overexpression has been associated with HCC progression and poor overall survival.

In contrast, LIG1 has not been extensively studied in HCC, despite its essential role in DNA replication and repair, particularly during lagging‐strand synthesis and base excision repair [33]. Its frequent upregulation in rapidly proliferating tumor cells aligns with its function in supporting high replication demand [34]. However, findings from a recent proteomic analysis by Chang et al. reported reduced LIG1 expression in recurrent HCC, suggesting dynamic regulation during progression or treatment response [35]. This complexity implies that while LIG1 may be overexpressed in primary tumors, its expression could decrease during recurrence, potentially reflecting shifts in DNA repair requirements. Although LIG1 has not yet been validated as a clinical biomarker in HCC, its essential function and elevated expression in other malignancies, including breast and ovarian cancers, underscore its potential therapeutic relevance [36].

Taken together, these findings support the clinical significance of both IRAK1 and LIG1 in HCC. Our results reinforce the role of IRAK1 as a validated diagnostic and prognostic biomarker in HCC [37]. In contrast, LIG1 emerges as a candidate diagnostic biomarker, but not a prognostic one: unlike IRAK1, its association with survival was not independent of tumor stage and other clinicopathological covariates in multivariate analysis (HR = 1.02, 95% CI: 0.76–1.35, *p* = 0.916). Its essential role in HCC cell viability identifies it as a candidate therapeutic dependency rather than a validated therapeutic target. Further experimental and clinical validation is required to determine whether targeting LIG1 could contribute to improved patient outcomes.

In contrast to the oncogenic genes identified, ADH4 was significantly downregulated at both mRNA and protein levels, with low expression strongly linked to poor survival (*p* = 0.00045). Although ADH4 was not essential for HCC cell viability in DepMap analysis, its consistent downregulation supports a tumor‐suppressive role. This is supported by Wei et al. who identified reduced ADH4 as an independent prognostic marker in HCC [38], and more recent studies linking ADH4 to TP53‐related prognostic signatures and immune/metabolic regulation [39]. Mechanistically, ADH4 contributes to retinoic acid production, which suppresses Wnt/β‐catenin signaling, and its restoration has been shown to inhibit tumor growth and enhance chemotherapy sensitivity [40]. In our study, ADH4 emerged as a validated diagnostic and prognostic biomarker in HCC, consistent with previous reports demonstrating its value as a clinically relevant marker for HCC diagnosis and prognosis [41]. Although TERT, IRAK1, and ADH4 have known roles in liver biology, their combined analysis may improve prognostic accuracy. Further validation is needed to confirm their clinical utility, and other identified genes warrant deeper investigation due to their potential role in HCC heterogeneity.

The pan‐cancer expression analysis of LIG1, MSTO1, TERT, IRAK1, and ADH4 provides insights into their potential functional relevance in HCC. The consistent upregulation of LIG1, MSTO1, and IRAK1 in HCC and multiple other malignancies suggests their involvement in general oncogenic processes, such as enhanced proliferation, metabolic adaptation, and inflammatory signaling. Their non‐specific expression profiles indicate that while they may contribute to hepatocarcinogenesis, they are not exclusive to liver cancer and may reflect broader tumor‐associated pathways. In contrast, TERT demonstrated a more restricted expression pattern, with strong upregulation in HCC and limited expression in normal tissues, supporting its recognized role as a key driver of telomerase reactivation in liver tumorigenesis. Notably, ADH4 showed a liver‐specific expression profile, with high levels in normal liver and significant downregulation in HCC, suggesting a potential tumor‐suppressive role and highlighting its utility as a biomarker for hepatobiliary cancers. These findings underscore the importance of distinguishing between broadly expressed oncogenes and tissue‐specific alterations.

Based on the cbioportal analysis, the high frequency of TERT promoter mutations underscores its pivotal role in HCC tumorigenesis through telomerase activation and enhanced cellular immortality. The relatively low mutation rates in MSTO1 and LIG1 suggest that their oncogenic contributions are not primarily mutation‐driven but may arise from transcriptional upregulation or post‐transcriptional regulation. Similarly, the limited genomic alterations observed in IRAK1 and ADH4 imply that their involvement in HCC could be linked to signaling or epigenetic mechanisms rather than direct somatic mutations. Overall, while TERT represents a genomically altered hallmark in HCC, the other studied genes likely contribute through non‐mutational regulatory pathways, reflecting the molecular heterogeneity underlying hepatocarcinogenesis. These results were confirmed by the promoter methylation analysis of MSTO1 and ADH4 using the UALCAN portal.

The correlation of MSTO1 and LIG1 expressions with different immune infiltrates may provide a basis for predicting immunotherapy response. Both MSTO1 and LIG1 expressions were positively correlated with the estimated infiltration of M0 macrophages, activated NK cells, NK‐like T cells, and Tregs, and inversely associated with M2 macrophages. However, they differed regarding CD8^+^ central memory T cells: MSTO1 showed a significant negative association, whereas the correlation for LIG1 was not statistically significant. Their positive correlation with cytotoxic effectors (NK cells) and M0 macrophages, and negative correlation with pro‐tumorigenic M2 macrophages, indicate a functional shift in the HCC microenvironment which may favor anti‐tumor immunity. Moreover, the negative correlation with Tregs creates a complex interaction whereby tumor‐intrinsic gene expression may lead to immune activation and a compensatory immunosuppression. Due to the differential response of HCC patients to immune checkpoint inhibitors (ICIs), MSTO1 and LIG1 can be considered as promising candidates for multigene predictive signatures requiring validation in ICI‐treated cohorts rather than standalone predictive biomarkers.

Despite the valuable insights gained from in silico analyses, this study has several limitations. Reliance on publicly available datasets, such as TCGA, may introduce sample bias, platform variability, and technical artifacts (e.g., sequencing bias, uneven coverage). Additionally, bulk RNA‐seq cannot resolve tumor heterogeneity, limiting distinction between cancer cells, stromal components, and immune infiltrates.

Although the study confirms protein expression levels using both CPTAC and HPA data, there are significant inconsistencies for some markers, notably MSTO1 and GHR. This could be attributed to the differences in methodologies of these two databases. CPTAC database uses the proteomics mass spectrometry technology that gives quantitative high‐throughput data via peptide identification, whilst the HPA database uses the immunohistochemical (IHC) staining, a semi‐quantitative method sensitive to antibody specificity. The HPA staining method is a good technique with excellent spatial resolution. However, its reliability is affected by factors such as antibody specificity, detection procedures, and tissue heterogeneity. Moreover, the clinical relevance of the HPA data used in this research is limited due to the smaller sample size. Therefore, although these results provide an overview of differential protein expression, they should be considered preliminary. Further validation of these biomarkers in larger independent patient cohorts is required to support strong clinical conclusions, particularly where results from HPA and CPTAC diverge, to establish robust diagnostic and prognostic confidence.

While tools like TCGA, CPTAC, DepMap, and Kaplan–Meier Plotter offer predictive insights, experimental validation remains essential to confirm the roles of candidate genes such as MSTO1 and LIG1. The heterogeneity of HCC, driven by diverse etiologies (HBV, HCV, alcohol) and genetic backgrounds, also necessitates further context‐specific investigation.

Future studies should include functional validation via CRISPR/Cas9, siRNA knockdown, drug sensitivity assays, and IHC in larger, clinically annotated cohorts. Incorporating these genes into multi‐marker panels may improve early detection and enhance the diagnostic and prognostic accuracy of HCC biomarkers.

## 5 Conclusion

Our study identified LIG1 and MSTO1 as candidate diagnostic biomarkers in hepatocellular carcinoma (HCC). Notably, MSTO1 demonstrated excellent diagnostic discrimination (AUC = 0.987), a significant prognostic association after adjusting for baseline clinicopathological factors (but not after adding AFP status), and a potential therapeutic dependency. LIG1 showed good diagnostic discrimination (AUC = 0.897) but no independent prognostic association in either multivariate model. Established biomarkers, TERT, ADH4, and IRAK1, were validated, with TERT showing strong diagnostic and therapeutic dependency potential, ADH4 retaining diagnostic and prognostic value, and IRAK1 confirmed as a reliable diagnostic and prognostic marker. Following Benjamini‐Hochberg FDR correction, ADH4, LIG1, IRAK1, and MSTO1 retained significant associations with survival, whereas GHR, ASPG, TERT, and HBB did not.

## Author Contributions

**Jana Hamad:** conceptualization, methodology, data curation, supervision, investigation, writing – original draft, writing – review and editing. **Hussein Fayyad‐Kazan:** conceptualization, methodology, data curation, investigation, writing – original draft, writing – review and editing, supervision. **Fadi Abdel‐Sater:** conceptualization, methodology, data curation, supervision, writing – review and editing, writing – original draft, investigation.

## Funding

The authors have nothing to report.

## Ethics Statement

All data analyzed in this study were obtained from publicly available sources. As these datasets are de‐identified and open‐access, the study did not require approval from an institutional ethics committee.

## Consent

The authors have nothing to report.

## Conflicts of Interest

The authors declare no conflicts of interest.

## Data Availability

The data supporting the findings of this study are openly available in public databases.
